# The immune factors have complex causal regulation effects on inflammatory bowel disease

**DOI:** 10.3389/fimmu.2023.1322673

**Published:** 2024-01-09

**Authors:** Binxu Qiu, Tao Zhang, Xinxin Qin, Shengjie Ma, Quan Wang

**Affiliations:** Department of Gastric and Colorectal Surgery, General Surgery Center, The First Hospital of Jilin University, Changchun, China

**Keywords:** inflammatory bowel disease, causal relationship, Mendelian randomization, Crohn’s disease, ulcerative colitis

## Abstract

**Background:**

Although a correlation between immune cell phenotypes and inflammatory bowel disease (IBD) has been established, a causal relationship remains unestablished.

**Methods:**

To assess causal associations between immune cell phenotypes and IBD and its subtypes, we employed Mendelian randomization (MR) methods and genome-wide association studies (GWAS) summary statistics. The primary outcomes were determined based on the inverse variance weighting (IVW) results, with the assessment of heterogeneity and pleiotropy conducted through Cochrane’s Q-test and MR-Egger. The stability of the MR results was then examined using leave-one-out analysis, and false discovery rate (FDR) correction was applied to evaluate the strength of the causal relationship between exposure and outcome. Furthermore, to identify immunophenotypes strongly associated with IBD, a meta-integration of the effect values of all positive results in both datasets was conducted.

**Results:**

The analysis of 731 immune cell phenotypes and IBD using MR techniques revealed potential causal associations between 26 phenotypes and IBD. Subsequent meta-integration of the two datasets provided evidence of solid causal associations between 18 immune phenotypes and IBD and its subtypes. Nominal causal associations were also identified in the remaining eight immune phenotypes and IBD and its subtypes.

**Conclusion:**

Our study confirms causal solid associations between 18 immune phenotypes and IBD, thus guiding future clinical studies.

## Introduction

Inflammatory bowel disease (IBD) is a chronic, multiple recurrent inflammatory disease of the gastrointestinal tract with two main subtypes: ulcerative colitis (UC) and Crohn’s disease (CD) (1). UC primarily involves the rectum and colon, whereas CD can affect any part of the gastrointestinal tract between the mouth and the anus. Despite their differences in terms of location of damage, symptoms, pathophysiology, course, and complications, they have in common the fact that they are usually diagnosed at an early age and may lead to a significant reduction in quality of life (2, 3). The exact pathogenesis of IBD is unknown; however, genetic predisposition, environmental factors, and immune system dysfunction are thought to play key roles in its development (4). IBD already affects more than 2.5 million Europeans, and its incidence is gradually increasing in Asia and developing countries, placing patients and healthcare systems (5). Therefore, early diagnosis and treatment of IBD is essential to control symptoms, improve prognosis, and reduce patient suffering (6). It is imperative to recognize the importance of early intervention to mitigate the impact of IBD on patients’ lives and to minimize the burden on healthcare systems.

Epidemiological studies have shown an association between infections in early adulthood and a variety of autoimmune-related diseases, including IBD, primary biliary cirrhosis, rheumatoid arthritis and systemic lupus erythematosus (7–9). The pathogenesis and treatment of these diseases are associated with complex interactions between gut health and the immune system, and therefore a deeper understanding of these interrelationships is important for our understanding of disease pathogenesis and therapeutic potential. Cytokines play a key role in infection and inflammation, and they are key mediators of interactions between the gut and the immune system (10, 11). However, findings on the association between immune inflammation and IBD have been inconsistent, possibly due to several factors such as limited sample sizes, flawed study designs, and failure to account for confounding factors.

Mendelian randomization (MR) is an emerging method of epidemiological analysis that uses genetic variation as a tool to assess causal relationships between exposures and clinical outcomes (12, 13). The method is founded on the principle of random distribution in biology, which makes its results immune to potential confounders and reverse causation. Because the distribution of genetic variation is randomized across generations, the MR approach can provide strong evidence to support the causal role of risk factors on outcomes. Previous studies have identified multiple associations between immune cell profiles and IBD, providing preliminary evidence for a causal relationship between them (14, 15). In this study, we employed a two-sample MR approach to determine the causal relationship between immune phenotypes and IBD and its subtypes. By utilizing this approach, we aim to contribute to a deeper understanding of the pathogenesis of IBD and provide potential therapeutic avenues.

## Materials and methods

### Study design

**Figure 1:**
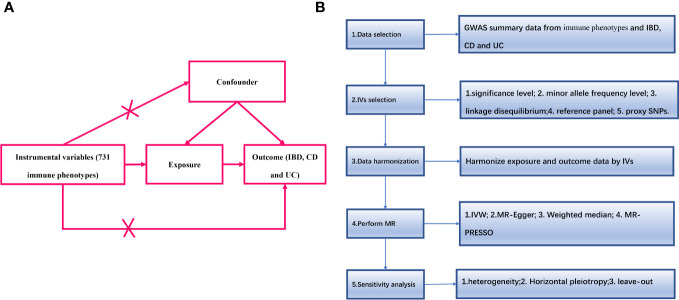
**(A)** The diagram of MR assumption; **(B)** Diagram of MR analysis processing. SNPs, single nucleotide polymorphisms; GWAS, genome-wide association study; MR, Mendelian randomization; IBD, Inflammatory Bowel Disease; CD, Crohn’s disease; UC, ulcerative colitis; IVs instrumental variables; IVW, inverse variance weighting; MR-Egger, MR-Egger regression; MR-PRESSO, Mendelian Randomization Pleiotropy RESidual Sum and Outlier (MR-PRESSO) test.

An overview of the study design is shown in **Figure 1**. We used two-sample MR analyses to assess causal associations between 731 immune phenotypes and IBD and its subtypes. In the MR analyses, we used genetic variation as a proxy for risk factors, and therefore, such causal inference relies on valid instrumental variables (IVs) that must satisfy three key assumptions (16):(1) genetic variation is directly associated with exposure factors; (2) genetic variation is independent of confounders that may influence the relationship between exposure and outcome; and (3) genetic variation does not influence outcome through other than exposure pathways that affect outcomes.

### IBD’s data source for genome-wide association study

To comprehensively determine the causal relationship between immune phenotypes and IBD and its subtypes, the study used IBD data from two major data sources. Firstly, we used the largest IBD GWAS data published by the IBD Genetics Consortium (IBDGC), which includes UC and CD (17). The complete IBD (units, logOR) GWAS summary statistics can be downloaded from the International IBDGC website (https://www.ibdgenetics.org/downloads.html). These data summarize the largest IBD GWAS, including IBD (N = 12882 cases, 21770 controls), UC (N = 6968 cases, 20464 controls) and CD (N = 5956 cases, 14927 controls) (17). Secondly, we also included GWAS data from another larger IBD study, which is available from the IEU GWAS database (https://gwas.mrcieu.ac.uk/datasets/). All study participants were of European ancestry. To confirm our findings, we also performed an integrative analysis including IBD 25042 cases and 34915 controls, as well as statistical data from immunoassay full association analyses for UC (N = 12366 cases, 33609controls) and CD (N = 12194 cases, 28072 controls) (18). To minimize the potential impact of population stratification, all participants were of European ancestry. The GWAS summary statistics used in our study can be downloaded from the official website (https://gwas.mrcieu.ac.uk/datasets/). All these data have been de-identified and are free to download and can be used freely. Details of phenotypes were shown in **Table 1**.

**Table 1:** Information of GWAS summary data.

Characteristic	Resource	Sample size	Population	PMID
IBD (IEU)	MRC-IEU	25042 cases and 34915 controls	European	PMID: 28067908
CD (IEU)	MRC-IEU	12194 cases and 28072 controls	European	PMID: 28067908
UC (IEU)	MRC-IEU	12366 cases and 33609controls	European	PMID: 28067908
IBD (IBDGC)	IBDGC	12882 cases and 21770 controls	European	PMID:26192919
CD (IBDGC)	IBDGC	5956 cases and 14927 controls	European	PMID: 26192919
UC (IBDGC)	IBDGC	6968 cases and 20464controls	European	PMID:26192919
immune phenotypes	GWAS catalogue	3757 cases	European	PMID:32929287

IBD, Inflammatory Bowel Disease; CD, Crohn’s disease; UC, ulcerative colitis; IBDGC, The International Inflammatory Bowel Disease Genetics Consortium; IEU, MRC Integrative Epidemiology Unit; GWAS, genome-wide association study.

### Immune cell-related GWAS data sources

GWAS summary statistics for each immunological characteristic are publicly available in the GWAS catalogue (archive numbers from GCST0001391 to GCST0002121) (19). A total of 731 different immunophenotypes were included, including absolute cell counts (AC), median fluorescence intensities (MFI), morphological parameters (MP), and relative cell counts (RC). Specifically, MFI, AC, and RC included various immune cell types such as B cells, Dendritic Cell, different T cell maturation stages, monocytes (MC), myeloid cells, and Treg panels. In contrast, the MP function includes the Dendritic Cell and TBNK panels. The raw GWAS data for these immune characteristics were based on information from 3,757 European individuals and did not involve overlapping data cohorts. Genotyping of approximately 240,000 single nucleotide polymorphisms (SNPs) was performed using a reference panel based on Sardinian sequences, followed by association testing after correction for covariates (sex, age and year) (19).

### Screening of IVs

The selection criteria for the IVs were as follows:(1) we firstly selected those SNPs associated with immune cell phenotypes at the genome-wide significance threshold (P < 1.0 × 10-5) as potential IVs (20); (2) using data from the European samples in the 1000 Genomes Project as a reference panel, we computed the SNPs between the linkage disequilibrium (LD), filtering out SNPs with R2 < 0.001 (Lumped window size = 10,000 kb) and retaining only those with the lowest P-values; (3) we excluded SNPs with a minor allele frequency (MAF) of ≤ 0.01; (4) when alleles were present, we used the allele frequency information to infer orthologous linkage alleles; and (5) to ensure a strong association with the exposure factor associations, we selected as IVs those SNPs with F-statistic values greater than 10. The F-statistic was calculated as F = Beta^2^/SE^2^.

### MR analysis

To ensure that our analyses were more robust, this study used several methods to assess the causal relationship between IBD (including CD and UC) and multiple immune cell phenotypes, including the inverse variance weighting (IVW) method, the weighted median (WM) method, MR-Egger regression, and MR-Polytomous RESidual Sum and Outliers (MR-PRESSO) method. The primary method of analysis was the IVW method, which used a meta-analytic approach to pool the Wald estimates for each SNP to obtain an overall estimate of the effect on the association between immune cell subtypes and IBD (21). If horizontal pleiotropy is not present, then unbiased causal estimates can be obtained by IVW linear regression (22). In addition, MR-Egger regression provides a weighted linear regression of the outcome coefficients based on the InSIDE assumption (23), which provides a valid test of the null hypothesis of causality and a consistent estimate of the causal effect even if all of the genetic variants are null IVs. However, MR-Egger estimates may not be accurate enough and are susceptible to strong effects of peripheral genetic variation. To avoid the InSIDE assumption, WM estimation is superior to MR-Egger because of its better performance in causal effect detection and type I error control (24). In addition, the MR-PRESSO method is a powerful tool for excluding horizontal polytropic outliers that may have a serious impact on the estimation results (25). To present a comprehensive and accurate picture of the causal relationship between immune cell subtypes and IBD (including CD and UC), we used the false discovery rate (FDR) correction to fine-tune the causal link between the two. We also used the odds ratio (OR) and 95% confidence interval (CI) to assess the relative risk between IBD (including CD and UC) and immune cell subtypes. Finally, to obtain comprehensive results and maintain consistency of results, we meta-integrated the results from two datasets. We integrated the results using random effects meta results if I^2^ >50% and fixed effects meta results if I^2^ ≤50%. We categorized the results into strong and nominal causal associations, based on whether they remained positive after meta-integration. The STROBE-MR guidelines were used to guide the design of this study (26). All statistical analyses and data visualization were performed in R software 3.4.0 (https://www.r-project.org/) using several R packages (including TwoSampleMR, forestploter, meta and dplyer).

## Result

### Causal effects of immune phenotypes on IBD

**Figure 2:**
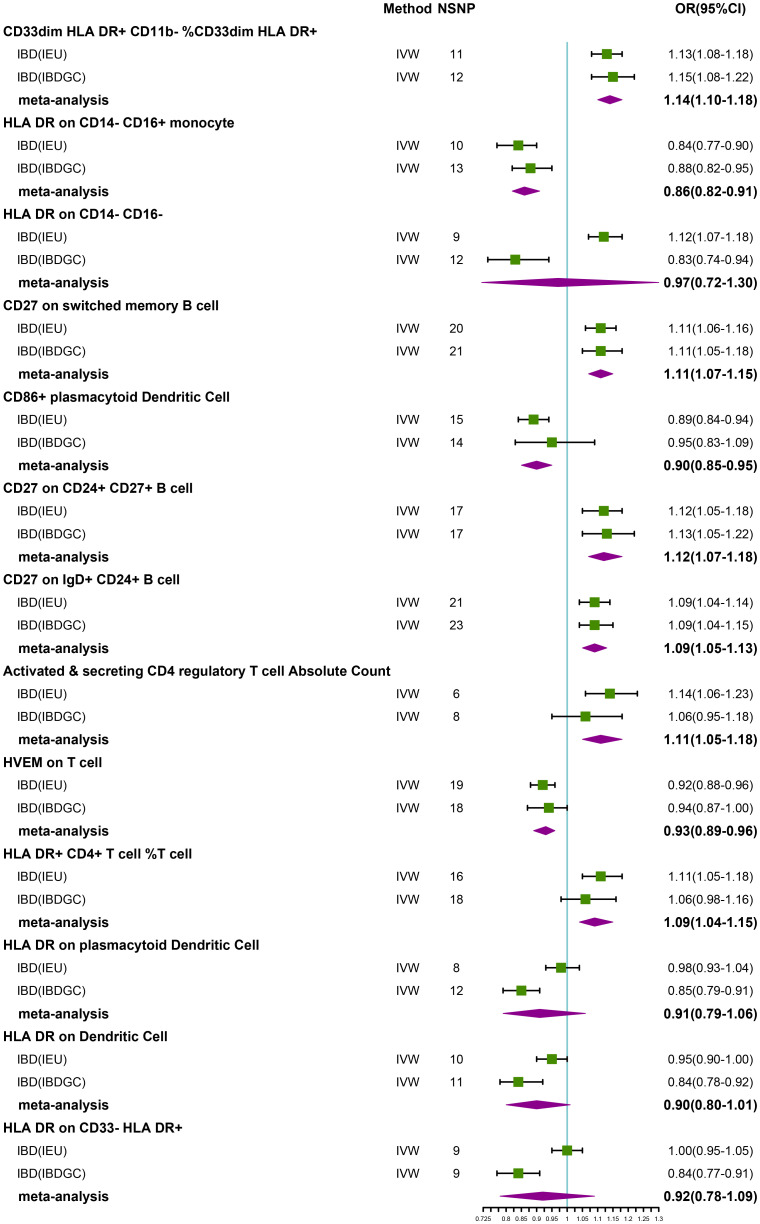
Forest plot of meta-analysis of causal estimation of immune phenotypes for IBD. NSNP, Number of SNPs; OR, odds ratio; CI, confidence interval of OR; IVW, inverse variance weighting; IBD, Inflammatory Bowel Disease; IBDGC, The International Inflammatory Bowel Disease Genetics Consortium; IEU, MRC Integrative Epidemiology Unit; HLA, human leukocyte antigen; FDR, false discovery rate.

After FDR correction, we identified 13 potential causal relationships in two datasets. In the IEU cohort, IVW results showed genetically predicted CD33dim HLA DR+ CD11b- %CD33dim HLA DR+ myeloid cell (OR: 1.13, 95% CI: 1.08–1.18, *P=1.00 × 10^−8^*), HLA DR on CD14- CD16- MC (OR:1.12, 95% CI: 1.07–1.18, *P=1.08 × 10^−5^*), CD27 on switched memory B cell (OR: 1.11, 95% CI: 1.06–1.16, *P=3.15 × 10^−5^*), CD27 on CD24+ CD27+ B cell (OR: 1.12, 95% CI: 1.05–1.18, *P=1.47 × 10^−4^*), CD27 on IgD+ CD24+ B cell (OR: 1.09, 95% CI: 1.04–1.14, *P=2.25 × 10^−4^*), Activated & secreting CD4 regulatory T cell AC (OR: 1.14, 95% CI: 1.06–1.23, *P=3.33 × 10^−4^*), and HLA DR+ CD4+ T cell %T cell (OR: 1.11, 95% CI: 1.05–1.18, *P=5.90 × 10^−4^*) were elevated may increase the risk of IBD (**Table 2**; **Figure 2**). Furthermore, we found that genetically predicted HLA DR on CD14- CD16+ MC (OR: 0.84, 95% CI: 0.77–0.90, *P=4.12 × 10^−6^*), CD86+ plasmacytoid Dendritic Cell %Dendritic Cell (OR: 0.89, 95% CI: 0.84–0.94, *P=5.01 × 10^−5^*) and HVEM on T cell (OR: 0.92, 95% CI: 0.88–0.96, *P=4.55 × 10^−4^*) elevation reduced the risk of IBD (**Table 2**; **Figure 2**). The P-value of the MR-Egger intercept was higher than 0.05, indicating that no significant pleiotropy was found (**Table 2**). Meanwhile, The β-values of the MR-Egger and WM analysis results were in the same direction as the IVW results, reinforcing the robustness of our findings (**Table 2**). Although Cochrane’s Q test indicated heterogeneity in some positive results, our findings remain reliable due to the use of IVW results with random effects (27) (*P<0.05*, **Table 2**). Detailed information from sensitivity analyses demonstrated the robustness of the observed causal associations (**Supplementary Table S1**). In the IBDGC cohort, we found genetically predicted CD27 on IgD+ CD24+ B cell (OR: 1.09, 95% CI: 1.04-1.14, *P=1.76× 10^-4^*), CD27 on CD24+ CD27+ B cell (OR: 1.13, 95% CI: 1.06–1.21, *P=3.18× 10^-4^*), CD27 on switched memory B cell (OR: 1.11, 95% CI: 1.05–1.18, *P=3.62× 10^-4^*) and CD33dim HLA DR+ CD11b- %CD33dim HLA DR+ myeloid cell (OR: 1.15, 95% CI: 1.08–1.22, *P=1.17× 10^−5^*) can increase the risk of IBD (**Table 2**; **Figure 2**). Meanwhile, we found that genetic HLA DR on plasmacytoid Dendritic Cell (OR: 0.85, 95% CI: 0.79–0.91, *P=2.67 × 10^−6^*), HLA DR on Dendritic Cell (OR: 0.84, 95% CI: 0.78–0.92, *P=7.59 × 10^−5^*) and HLA DR on CD33- HLA DR+ (OR: 0.84, 95% CI: 0.77–0.91, *P=6.82 × 10^−5^*) were elevated to reduce the risk of IBD (**Table 2**; **Figure 2**). Despite some positive results exhibiting heterogeneity according to Cochrane’s Q test, our findings remain reliable due to the use of IVW results with random effects (27) (*P<0.05*, **Table 2**). The β-values from the MR-Egger and WM analyses were consistent with the IVW results, supporting the strength of our findings (**Table 2**). Additionally, the MR-Egger intercept’s P-value exceeded 0.05, suggesting an absence of significant pleiotropy (**Table 2**). Detailed information from sensitivity analyses demonstrated the robustness of the observed causal associations (**Supplementary Table S2**). Scatterplots and funnel plots also demonstrated the stability of the results (**Supplementary Figures S1**, **S2**). Meta-integration of IVW results from both cohorts indicated that nine genetically predicted immunophenotypes remained strongly causally associated with IBD, and that genetically predicted HLA DR on plasmacytoid Dendritic Cell, HLA DR on Dendritic Cells, HLA DR on CD33- HLA DR+ and HLA DR on CD14- CD16- had nominal causal associations with IBD (**Figure 2**). Additionally, leave-one-out analyses showed that no single IV resulted in the identified causal associations (**Supplementary Figures S1**, **S2**).

**Table 2:** Positive MR results of causal links between immune cells phenotypes and IBD.

Exposure	Outcome	Method	β	NSNP	Pval	OR	LCI	UCI	Cochran’s Q	P_Cochran’s Q	P_Egger-intercept
CD33dim HLA DR+ CD11b- %CD33dim HLA DR+	IBD (IEU)	IVW	0.12	11	1.00E-08	1.13	1.08	1.18	18.82	4.26E-02	5.03E-02
	IBD (IEU)	MR-Egger	0.21	11	2.33E-04	1.24	1.15	1.33	10.02	3.49E-01	NA
	IBD (IEU)	WM	0.15	11	2.29E-10	1.17	1.11	1.22	NA	NA	NA
HLA DR on CD14- CD16-	IBD (IEU)	IVW	0.12	9	1.08E-05	1.12	1.07	1.18	6.14	6.31E-01	2.73E-01
	IBD (IEU)	MR-Egger	0.19	9	2.36E-02	1.21	1.06	1.37	4.73	6.93E-01	NA
	IBD (IEU)	WM	0.13	9	2.38E-04	1.14	1.06	1.22	NA	NA	NA
CD27 on switched memory B cell	IBD (IEU)	IVW	0.10	20	3.15E-05	1.11	1.06	1.16	50.35	1.16E-04	6.64E-01
	IBD (IEU)	MR-Egger	0.12	20	3.34E-02	1.13	1.02	1.26	49.81	8.06E-05	NA
	IBD (IEU)	WM	0.11	20	2.03E-06	1.11	1.06	1.16	NA	NA	NA
CD27 on CD24+ CD27+ B cell	IBD (IEU)	IVW	0.11	17	1.47E-04	1.12	1.05	1.18	49.08	3.21E-05	5.00E-01
	IBD (IEU)	MR-Egger	0.08	17	2.16E-01	1.08	0.96	1.21	47.56	2.99E-05	NA
	IBD (IEU)	WM	0.11	17	3.52E-07	1.12	1.07	1.17	NA	NA	NA
CD27 on IgD+ CD24+ B cell	IBD (IEU)	IVW	0.08	21	2.25E-04	1.09	1.04	1.14	34.83	2.10E-02	8.35E-01
	IBD (IEU)	MR-Egger	0.09	21	5.41E-02	1.10	1.00	1.20	34.75	1.50E-02	NA
	IBD (IEU)	WM	0.11	21	5.59E-05	1.11	1.06	1.17	NA	NA	NA
Activated & secreting CD4 regulatory T cell Absolute Count	IBD (IEU)	IVW	0.13	6	3.33E-04	1.14	1.06	1.23	3.70	5.94E-01	9.09E-01
	IBD (IEU)	MR-Egger	0.15	6	2.04E-01	1.16	0.96	1.40	3.68	4.51E-01	NA
	IBD (IEU)	WM	0.17	6	8.22E-04	1.19	1.07	1.32	NA	NA	NA
HLA DR+ CD4+ T cell %T cell	IBD (IEU)	IVW	0.10	16	5.90E-04	1.11	1.05	1.18	18.05	2.60E-01	6.311E-02
	IBD (IEU)	MR-Egger	0.26	16	3.21E-03	1.30	1.13	1.51	12.68	5.52E-01	NA
	IBD (IEU)	WM	0.12	16	6.54E-03	1.12	1.03	1.22	NA	NA	NA
HLA DR on CD14- CD16+ monocyte	IBD (IEU)	IVW	-0.18	10	4.12E-06	0.84	0.77	0.90	28.34	8.38E-04	5.29E-02
	IBD (IEU)	MR-Egger	-0.34	10	2.29E-03	0.71	0.61	0.83	17.23	2.78E-02	NA
	IBD (IEU)	WM	-0.16	10	1.98E-06	0.85	0.79	0.91	NA	NA	NA
CD86+ plasmacytoid Dendritic Cell %Dendritic Cell	IBD (IEU)	IVW	-0.11	15	5.01E-05	0.89	0.84	0.94	12.20	5.90E-01	8.70E-01
	IBD (IEU)	MR-Egger	-0.13	15	2.08E-01	0.88	0.72	1.06	12.17	5.13E-01	NA
	IBD (IEU)	WM	-0.09	15	1.76E-02	0.91	0.84	0.98	NA	NA	NA
HVEM on T cell	IBD (IEU)	IVW	-0.08	19	4.55E-04	0.92	0.88	0.96	38.29	3.55E-03	7.10E-01
	IBD (IEU)	MR-Egger	-0.05	19	5.51E-01	0.95	0.80	1.12	37.97	2.48E-03	NA
	IBD (IEU)	WM	-0.06	19	2.89E-02	0.95	0.90	0.99	NA	NA	NA
HLA DR on plasmacytoid Dendritic Cell	IBD (IBDGC)	IVW	-0.17	12	2.67E-06	0.85	0.79	0.91	44.57	5.78E-06	8.59E-02
	IBD (IBDGC)	MR-Egger	-0.23	12	4.93E-04	0.80	0.73	0.87	32.70	3.06E-04	NA
	IBD (IBDGC)	WM	-0.21	12	4.23E-21	0.81	0.78	0.85	NA	NA	NA
CD33dim HLA DR+ CD11b- %CD33dim HLA DR+	IBD (IBDGC)	IVW	0.14	15	1.17E-05	1.15	1.08	1.22	32.69	3.19E-03	5.31E-02
	IBD (IBDGC)	MR-Egger	0.26	15	6.40E-04	1.30	1.16	1.45	22.75	4.48E-02	NA
	IBD (IBDGC)	WM	0.16	15	1.05E-05	1.18	1.09	1.27	NA	NA	NA
HLA DR on CD33- HLA DR+	IBD (IBDGC)	IVW	-0.18	9	6.82E-05	0.84	0.77	0.91	39.49	3.99E-06	7.48E-02
	IBD (IBDGC)	MR-Egger	-0.27	9	2.33E-03	0.76	0.68	0.85	24.31	1.01E-03	NA
	IBD (IBDGC)	WM	-0.17	9	2.72E-05	0.84	0.78	0.91	NA	NA	NA
HLA DR on Dendritic Cell	IBD (IBDGC)	IVW	-0.17	11	7.59E-05	0.84	0.78	0.92	49.14	3.83E-07	5.12E-03
	IBD (IBDGC)	MR-Egger	-0.29	11	9.13E-05	0.75	0.69	0.81	19.65	2.02E-02	NA
	IBD (IBDGC)	WM	-0.20	11	1.33E-12	0.82	0.77	0.86	NA	NA	NA
CD27 on IgD+ CD24+ B cell	IBD (IBDGC)	IVW	0.09	24	1.76E-04	1.09	1.04	1.14	25.19	3.41E-01	3.46E-01
	IBD (IBDGC)	MR-Egger	0.13	24	1.23E-02	1.14	1.04	1.24	24.17	3.38E-01	NA
	IBD (IBDGC)	WM	0.13	24	1.20E-04	1.14	1.07	1.22	NA	NA	NA
CD27 on CD24+ CD27+ B cell	IBD (IBDGC)	IVW	0.12	21	3.18E-04	1.13	1.06	1.21	49.01	3.07E-04	5.67E-01
	IBD (IBDGC)	MR-Egger	0.09	21	2.04E-01	1.09	0.96	1.25	48.15	2.44E-04	NA
	IBD (IBDGC)	WM	0.12	21	3.85E-05	1.13	1.07	1.20	NA	NA	NA
CD27 on switched memory B cell	IBD (IBDGC)	IVW	0.11	22	3.62E-04	1.11	1.05	1.18	52.60	1.57E-04	6.92E-01
	IBD (IBDGC)	MR-Egger	0.13	22	4.53E-02	1.14	1.01	1.28	52.18	1.07E-04	NA
	IBD (IBDGC)	WM	0.12	22	6.14E-05	1.12	1.06	1.19	NA	NA	NA

IBD, Inflammatory Bowel Disease; IBDGC, The International Inflammatory Bowel Disease Genetics Consortium; IEU, MRC Integrative Epidemiology Unit; GWAS, genome-wide association study; IVW, inverse variance weighting; HLA, human leukocyte antigen; NSNP, Number of SNPs; WM, weighted median; MR, Mendelian randomization; OR, odds ratio; UCI, upper confidence interval; LCI, lower confidence interval, and NA, not applicable.

### Causal effects of immune phenotypes on CD

**Figure 3:**
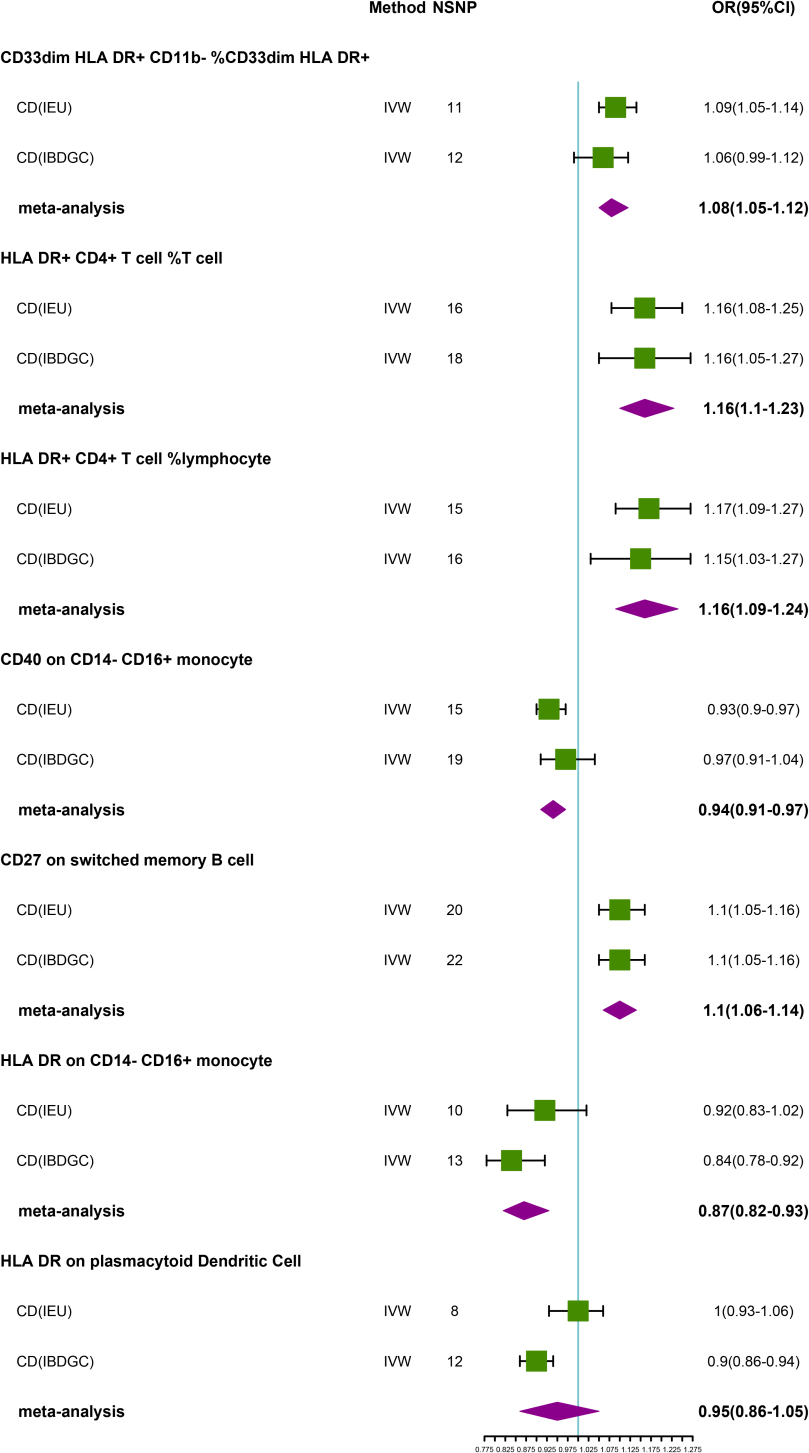
Forest plot of meta-analysis of causal estimation of immune phenotypes for CD. NSNP, Number of SNPs; OR, odds ratio; CI, confidence interval of OR; IVW, inverse variance weighting; CD, Crohn’s disease; IBDGC, The International Inflammatory Bowel Disease Genetics Consortium; IEU, MRC Integrative Epidemiology Unit; HLA, human leukocyte antigen; FDR, false discovery rate.

To explore the causal association of immunophenotypes on CD, we performed MR analysis with the IVW method as the primary analysis. After adjusting based on the FDR method, we identified five potential causal associations in the IEU cohort. We found genetically predicted CD33dim HLA DR+ CD11b- %CD33dim HLA DR+ myeloid cell (OR: 1.09, 95% CI: 1.05–1.14, *P=1.68 × 10^−5^*), HLA DR+ CD4+ T cell %T cell (OR: 1.16, 95% CI: 1.08–1.25, *P=4.40 × 10^−5^*), HLA DR+ CD4+ T cell % lymphocyte (OR: 1.17, 95% CI: 1.09–1.27, *P=5.05 × 10^−5^*) and CD27 on switched memory B cell (OR: 1.10, 95% CI: 1.05–1.16, *P=1.51 × 10^−4^*) may be associated with the development of CD (**Table 3**; **Figure 3**). Whereas, CD40 on CD14- CD16+ MC (OR: 0.93, 95% CI: 0.90–0.97, *P=7.62 × 10^−5)^* may reduce the occurrence of CD (**Table 3**; **Figure 3**). In the IBDGC cohort, we identified four potential causal associations. HLA DR on CD14- CD16+ MC (0.84, 95% CI: 0.78–0.92, *P=9.38× 10^−5^)* and HLA DR on plasmacytoid Dendritic Cell (OR: 0.90, 95% CI: 0.86–0.94, *P=1.27×10^-5^*) were associated with a lower incidence of CD (**Table 3**; **Figure 3**). However, CD27 on switched memory B cell (OR:1.10, 95% CI: 1.05–1.16, *P=1.97 × 10^−4^*) may elevate the occurrence of CD (**Table 3**; **Figure 3**). The combined analysis of IVW results from both cohorts revealed a strong and consistent causal association between the seven genetically predicted immunophenotypes and CD. There was also a suggestive causal link between HLA DR on plasmacytoid Dendritic Cell and CD (**Figure 3**). Our findings remain reliable due to the use of IVW results with random effects (*P<0.05*, **Table 3**), despite Cochrane’s Q test indicating heterogeneity in some positive results (27). The β-values from the MR-Egger and WM analyses were consistent with the IVW results, supporting the strength of our findings (**Table 3**). Moreover, the P-value for the MR-Egger intercept was above 0.05, indicating a lack of significant pleiotropy (**Table 3**). Detailed information from sensitivity analyses demonstrated the robustness of the observed causal associations (**Supplementary Tables S3**, **S4**). Scatterplots and funnel plots also demonstrated the stability of the results (**Supplementary Figure S3**, **S4**). In addition, the results of the leave-one-out analyses indicate that any single IV does not lead to the identified causal associations (**Supplementary Figures S3**, **S4**).

**Table 3:** Positive MR results of causal links between immune cells phenotypes and CD.

Exposure	Outcome	Method	NSNP	β	Pval	OR	LCI	UCI	Cochran’s Q	P_Cochran’s Q	P_Egger-intercept
HLA DR on plasmacytoid Dendritic Cell	CD (IBDGC)	IVW	12	-0.10	1.27E-05	0.90	0.86	0.94	8.80	6.40E-01	5.43E-01
	CD (IBDGC)	ME-Egger	12	-0.12	5.17E-03	0.89	0.83	0.95	8.40	5.89E-01	NA
	CD (IBDGC)	WM	12	-0.11	3.28E-05	0.90	0.85	0.94	NA	NA	NA
HLA DR on CD14- CD16+ monocyte	CD (IBDGC)	IVW	13	-0.17	9.38E-05	0.84	0.78	0.92	11.91	4.53E-01	3.53E-01
	CD (IBDGC)	ME-Egger	13	-0.27	3.52E-02	0.76	0.61	0.95	10.97	4.46E-01	NA
	CD (IBDGC)	WM	13	-0.19	1.11E-03	0.83	0.74	0.93	NA	NA	NA
CD27 on switched memory B cell	CD (IBDGC)	IVW	22	0.10	1.97E-04	1.10	1.05	1.16	20.08	5.16E-01	5.31E-01
	CD (IBDGC)	ME-Egger	22	0.12	2.33E-02	1.13	1.03	1.25	19.68	4.78E-01	NA
	CD (IBDGC)	WM	22	0.14	9.38E-05	1.15	1.07	1.23	NA	NA	NA
CD33dim HLA DR+ CD11b- %CD33dim HLA DR+	CD (IEU)	IVW	11	0.09	1.68E-05	1.09	1.05	1.14	8.88	5.44E-01	3.77E-01
	CD (IEU)	ME-Egger	11	0.05	2.89E-01	1.05	0.96	1.15	8.02	5.33E-01	NA
	CD (IEU)	WM	11	0.07	7.15E-03	1.07	1.02	1.13	NA	NA	NA
HLA DR+ CD4+ T cell %T cell	CD (IEU)	IVW	16	0.15	4.40E-05	1.16	1.08	1.25	16.01	3.82E-01	1.63E-01
	CD (IEU)	ME-Egger	16	0.28	1.09E-02	1.32	1.10	1.60	13.84	4.62E-01	NA
	CD (IEU)	WM	16	0.19	5.16E-04	1.20	1.08	1.34	NA	NA	NA
HLA DR+ CD4+ T cell %lymphocyte	CD (IEU)	IVW	15	0.16	5.05E-05	1.17	1.09	1.27	17.86	2.13E-01	5.55E-02
	CD (IEU)	ME-Egger	15	0.33	2.51E-03	1.39	1.17	1.66	13.32	4.23E-01	NA
	CD (IEU)	WM	15	0.19	5.52E-04	1.21	1.09	1.35	NA	NA	NA
CD27 on switched memory B cell	CD (IEU)	IVW	20	0.10	1.51E-04	1.10	1.05	1.16	33.73	1.98E-02	4.02E-01
	CD (IEU)	ME-Egger	20	0.14	2.12E-02	1.15	1.03	1.29	32.41	1.97E-02	NA
	CD (IEU)	WM	20	0.14	1.76E-07	1.15	1.09	1.22	NA	NA	NA
CD40 on CD14- CD16+ monocyte	CD (IEU)	IVW	15	-0.07	7.62E-05	0.93	0.90	0.97	9.49	7.98E-01	4.02E-01
	CD (IEU)	ME-Egger	15	-0.09	1.28E-02	0.91	0.85	0.97	8.74	7.92E-01	NA
	CD (IEU)	WM	15	-0.08	2.85E-04	0.93	0.89	0.97	NA	NA	NA

CD, Crohn’s disease; IBDGC, The International Inflammatory Bowel Disease Genetics Consortium; IEU, MRC Integrative Epidemiology Unit; GWAS, genome-wide association study; IVW, inverse variance weighting; HLA, human leukocyte antigen; NSNP, Number of SNPs; WM, weighted median; MR, Mendelian randomization. OR, odds ratio; UCI, upper confidence interval; LCI, lower confidence interval, and NA, not applicable.

### Causal effects of immune phenotypes on UC

**Figure 4:**
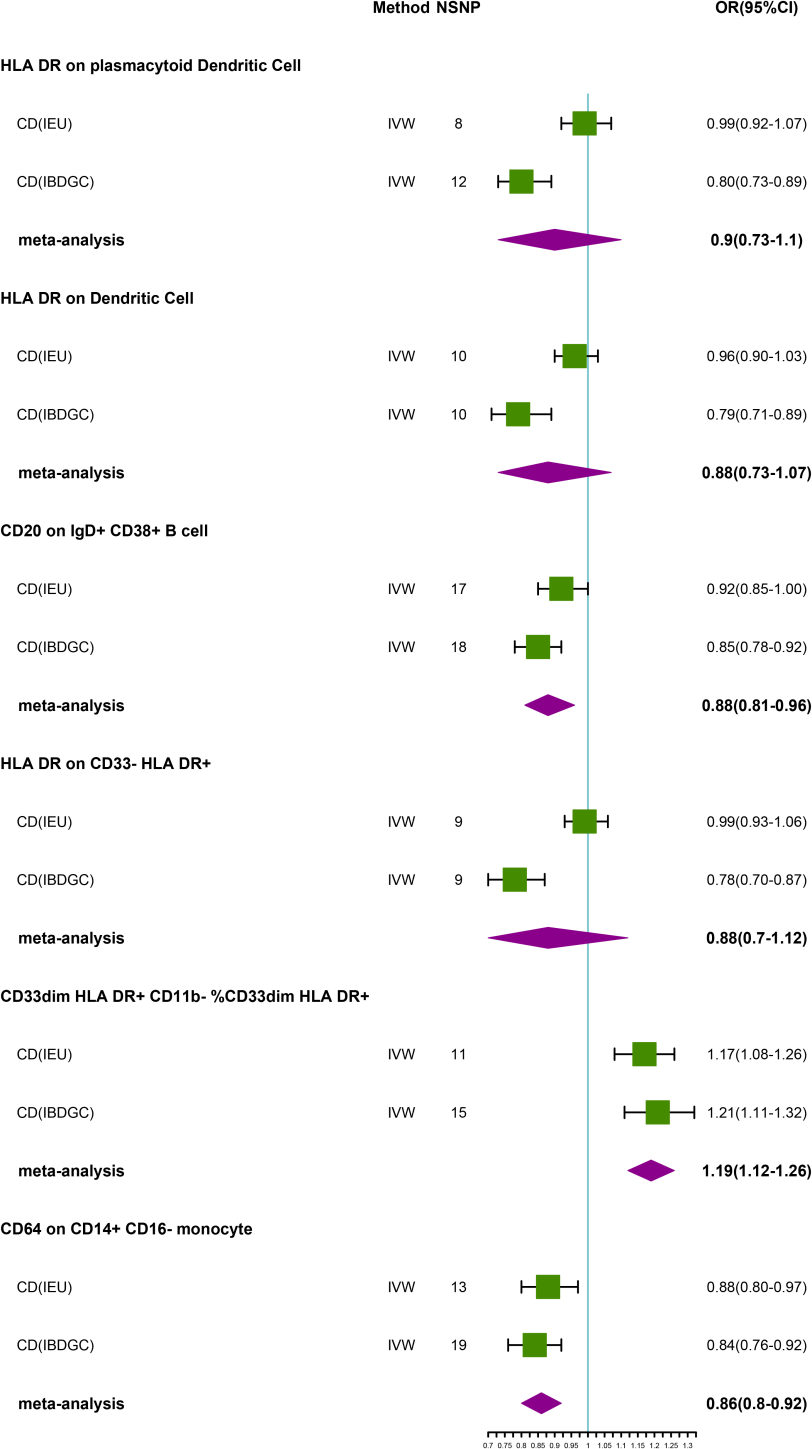
Forest plot of meta-analysis of causal estimation of immune phenotypes for UC. NSNP, Number of SNPs; OR, odds ratio; CI, confidence interval of OR; IVW, inverse variance weighting; UC, ulcerative colitis; IBDGC, The International Inflammatory Bowel Disease Genetics Consortium; IEU, MRC Integrative Epidemiology Unit; HLA, human leukocyte antigen; FDR, false discovery rate.

In the IEU cohort we did not find a causal association of immunophenotype on UC (**Supplementary Table S5**). However, in the IBDGC cohort, we identified six potential causal associations. HLA DR on plasmacytoid Dendritic Cell (OR: 0.80, 95% CI: 0.73–0.89, *P=3.70 × 10^−5^*), HLA DR on Dendritic Cell(OR: 0.79, 95% CI: 0.71–0.89, *P=5.88 × 10^−5^*), CD20 on IgD+ CD38+ B cell (OR: 0.85, 95% CI: 0.78–0.92, *P=1.20× 10^-4^)*, HLA DR on CD33- HLA DR+ (OR: 0.78, 95% CI:0.70–0.87, *P=1.32× 10^-5^*) and CD64 on CD14+ CD16- MC (OR: 0.84, 95% CI: 0.76–0.92, *P=1.71× 10^-4^, P_FDR_ =3.42× 10^−2^*) may reduce the incidence of UC (**Table 4**; **Figure 4**). CD33dim HLA DR+ CD11b- %CD33dim HLA DR+ myeloid cell (OR: 1.21, 95% CI: 1.11–1.32, *P=8.88× 10^−5^*) may increase the incidence of UC (**Table 4**; **Figure 4**). The MR-Egger intercept test suggests no significant pleiotropy (*P>0.05*, **Table 4**). Furthermore, the β-values from the MR-Egger and WM analyses were consistent with the IVW results, supporting the strength of our findings (**Table 4**). Despite heterogeneity in some positive results, as indicated by Cochrane’s Q test, our findings remain reliable as we utilized IVW results with random effects (P<0.05, **Table 4**) (27). Detailed information from sensitivity analyses demonstrated the robustness of the observed causal associations (**Supplementary Table S5**). Scatterplots and funnel plots also demonstrated the stability of the results (**Supplementary Figure S5**). In addition, the results of the leave-one-out analyses indicate that any single IV does not lead to the identified causal associations (**Supplementary Figure S5**). Meta-integration of IVW results from both cohorts showed that three genetically predicted immunophenotypes remained strongly causally associated with UC, with nominal causal associations between HLA DR on plasmacytoid Dendritic Cell, HLA DR on Dendritic Cell and HLA DR on CD33- HLA DR+ and UC (**Figure 4**).

**Table 4:** Positive MR results of causal links between immune cells phenotypes and UC.

Exposure	Outcome	Method	NSNP	β	Pval	OR	LCI	UCI	Cochran’s Q	P_Cochran’s Q	P_Egger-intercept
HLA DR on plasmacytoid Dendritic Cell	UC (IBDGC)	IVW	12	-0.22	3.70E-05	0.80	0.73	0.89	57.91	2.26E-08	5.07E-02
	UC (IBDGC)	ME-Egger	12	-0.32	4.44E-04	0.73	0.64	0.82	37.32	4.99E-05	NA
	UC (IBDGC)	WM	12	-0.27	4.23E-17	0.76	0.71	0.81	NA	NA	NA
HLA DR on Dendritic Cell	UC (IBDGC)	IVW	11	-0.23	5.88E-05	0.79	0.71	0.89	53.71	5.49E-08	1.26E-01
	UC (IBDGC)	ME-Egger	11	-0.41	2.08E-05	0.66	0.60	0.73	15.95	6.79E-02	NA
	UC (IBDGC)	WM	11	-0.25	1.98E-10	0.78	0.72	0.84	NA	NA	NA
CD20 on IgD+ CD38+ B cell HLA DR on CD33- HLA DR+	UC (IBDGC)	IVW	18	-0.17	1.20E-04	0.85	0.78	0.92	22.13	1.80E-01	8.18E-01
	UC (IBDGC)	ME-Egger	18	-0.14	3.57E-01	0.87	0.66	1.16	22.06	1.41E-01	NA
	UC (IBDGC)	WM	18	-0.16	5.38E-03	0.85	0.76	0.95	NA	NA	NA
HLA DR on CD33- HLA DR+	UC (IBDGC)	IVW	9	-0.25	1.32E-05	0.78	0.70	0.87	38.37	6.44E-06	5.415E-02
	UC (IBDGC)	ME-Egger	9	-0.40	3.11E-04	0.67	0.59	0.75	15.43	3.09E-02	NA
	UC (IBDGC)	WM	9	-0.36	4.40E-09	0.70	0.62	0.79	NA	NA	NA
CD64 on CD14+ CD16- monocyte	UC (IBDGC)	IVW	19	-0.18	1.71E-04	0.84	0.76	0.92	46.62	2.41E-04	2.62E-01
	UC (IBDGC)	ME-Egger	19	-0.33	3.80E-04	0.72	0.62	0.83	34.57	7.09E-03	NA
	UC (IBDGC)	WM	19	-0.10	6.49E-02	0.91	0.82	1.01	NA	NA	NA
CD33dim HLA DR+ CD11b- %CD33dim HLA DR+	UC (IBDGC)	IVW	15	0.19	7.82E-06	1.21	1.11	1.32	39.75	2.79E-04	1.23E-01
	UC (IBDGC)	ME-Egger	15	0.39	1.88E-04	1.47	1.27	1.71	24.09	3.03E-02	NA
	UC (IBDGC)	WM	15	0.13	1.88E-02	1.14	1.02	1.27	NA	NA	NA

UC, ulcerative colitis; IBDGC, The International Inflammatory Bowel Disease Genetics Consortium; IEU, MRC Integrative Epidemiology Unit; GWAS, genome-wide association study; IVW, inverse variance weighting; HLA, human leukocyte antigen; NSNP, Number of SNPs; WM, weighted median; MR, Mendelian randomization; OR, odds ratio; UCI, upper confidence interval; LCI, lower confidence interval, and NA, not applicable.

## Discussion

Despite our understanding that the immune system plays an essential role in autoimmune diseases, the immune system’s role in the development of IBD remains complex and needs to be clarified. The study is particularly noteworthy as it is the first MR analysis to use large-scale publicly available GWAS data as a genetic tool to investigate the potential causal relationship between multiple immune phenotypes and IBD (including CD and UC). The results of the study indicate that 26 immune phenotypes may be causally linked to IBD and its different subtypes. Combining the results from two datasets, we discovered that 18 immune phenotypes remain strongly causally linked to IBD and its subtypes. In contrast, the remaining six immune phenotypes are nominally causally associated with these diseases. Furthermore, our study demonstrates the significant involvement of immune phenotypes in this process, particularly among multiple subpopulations of MC, T cells, and B cells. In summary, these findings exceed our initial expectations regarding the role of immune factors in IBD. Importantly, our study shows no significant horizontal pleiotropy, which adds to the stability and reliability of these results.

Previous studies have shown a significant overlap in genetic susceptibility across various tissues in immune-mediated diseases, particularly IBD (28). Human leukocyte antigens (HLA) association suggests autoimmune involvement (29). Significantly, our findings reveal an increase in the relative counts of MC with the CD33dim HLA DR+ CD11b- phenotype in IBD (30). This effect is consistent across the classifications of IBD into CD and UC, indicating a general contribution of CD33dim HLA DR+ CD11b- MC to IBD (30). It is worth noting that CD33dim HLA DR+ CD11b- MC is a subset of myeloid cells characterized by low CD11b expression (30). CD11b, which is the alpha-chain of the integrin CR3, is predominantly found in MC neutrophils, natural killer (NK) cells, and other cell surfaces with high expression, and plays a crucial role in intra- and extracellular interactions through adhesion molecules (31). We propose that the mechanism by which CD33dim HLA DR+ CD11b- MC contributes to IBD involves the weakening of neutrophil adhesion function due to the absence of CD11b expression, and the induction of high levels of interleukin-1β(IL–1β), IL-6, and tumour necrosis factor-α (TNF-α) by MC (32).

CD14-CD16- HLA DR in the MC panel is associated with a reduced risk of IBD (**Figure 1**). HLA-DR, located on the cell surface, is an MHC II molecule encoded within the HLA complex at chromosome 6 region 6P21 (33). Previous research has suggested that reduced IL-1β concentrations in MC in IBD may point to diminished cell function (34). Evidence of low expression of HLA-DR in MC during chronic inflammation implies an anti-inflammatory role for HLA-DR molecules (33). Notably, while HLA-DR is typically linked to antigen-presenting cells, its expression on T cells is also observed and is associated with T cell activation. The underlying reasons and mechanisms behind activated T cells expressing HLA-DR remain somewhat ambiguous. A study by Tippalagama et al. investigating transcription analysis of CD4+ T cells from individuals with tuberculosis revealed that HLA-DR+ CD4 T cells in these individuals predominantly expressed phenotypic markers associated with effector memory phenotypes (CD45RA-CCR7-) and displayed increased expression of cytotoxic molecules and pro-inflammatory cytokines (34). This could provide a plausible explanation for the observed associations between HLA-DR+ CD4+ T cell %T cell and HLA DR+ CD4+ T cell %lymphocyte and IBD in our study. Hence, the expression of HLA-DR molecules on the surface of various types of immune cells may exert diverse effects in IBD, warranting further investigation into the underlying mechanisms.

Treg cells not only show competence in suppressing autoimmune diseases but also play a key role in controlling intestinal inflammation (35). In a mouse model of UC, a decrease in peripheral blood Treg cell numbers was observed (36). Nevertheless, it was found that increasing the secretion of IL-10 and TGF-β significantly improved diarrhoeal symptoms in mice (37). This indicates that Treg cells may mitigate the cascade and amplified response of intestinal inflammation by regulating the release of IL-10, TGF-β, and other anti-inflammatory factors, thus leading to an improvement in the clinical symptoms of IBD (38, 39).

The present study found a causal association between genetically related CD86 dendritic cells and a lower occurrence of CD. CD86 is a glycosylated protein of approximately 70 KDa consisting of 329 amino acids, a single transmembrane domain, and a cytoplasmic structural domain.CD86 is expressed by antigen-presenting cells, which are the target of CD28 and cytotoxic T-lymphocyte-associated protein 4 (CTLA-4) ligands (40). The binding of CD86 to CD28 is a co-stimulatory signal for the activation of T-cells, and the binding of CD86 to CTLA-4 negatively regulates T-cell activation and reduces immune responses (41). Thus, it is not difficult to understand that CD86 dendritic cells reduce the occurrence of CD. Herpesvirus invasion mediator (HVEM) is a recently intensively studied molecular switch widely expressed in hematopoietic and non-hematopoietic cells (42). HEVM can inhibit T-cells by binding to B- and T-lymphocyte attenuator (BTLA) without activating NK cells, which is probably why it reduces the incidence of IBD (43).

Although our study demonstrated a causal relationship between multiple immune phenotypes and IBD and its subtypes through MR analyses, there are several limitations that need to be acknowledged. Firstly, two-sample MR requires no sample overlap between exposure and outcome, but we encountered challenges in accurately estimating sample overlap. Nonetheless, we mitigated this issue by using a powered instrument (F-statistic >10) to minimize bias due to sample overlap (44). Secondly, the GWAS data utilized in our study was exclusively from the European population, necessitating caution in generalizing our findings to other populations. To address this limitation, we plan to conduct MR analyses on other populations to broaden and validate our results. Thirdly, we employed an FDR correction to evaluate the results, which may have resulted in some false negatives, potentially underestimating the causality of immune phenotypes on IBD and its subtypes. Furthermore, the lack of personal information limited our ability to conduct more comprehensive population stratification analyses. Finally, while our study demonstrated causal associations between multiple immune phenotypes and IBD, the precise biological mechanisms underlying these associations remain elusive. Consequently, additional functional studies are warranted to elucidate the impact of immune phenotypes on IBD.

## Conclusion

In summary, this study highlights a complex pattern of interactions between immunity and IBD. There is still a need to elicit the underlying mechanisms of the detected causal associations.

## Data availability statement

The original contributions presented in the study are included in the article/**Supplementary Material**. Further inquiries can be directed to the corresponding author.

## Author contributions

QB: Methodology, Software, Supervision, Writing – original draft, Writing – review & editing. TZ: Software, Writing – review & editing. XQ: Software, Writing – original draft. SM: Software, Writing – original draft. QW: Data curation, Software, Supervision, Writing – original draft, Writing – review & editing.
