# Corrigendum: The immune factors have complex causal regulation effects on inflammatory bowel disease

**DOI:** 10.3389/fimmu.2024.1368428

**Published:** 2024-01-23

**Authors:** Binxu Qiu, Tao Zhang, Xinxin Qin, Shengjie Ma, Quan Wang

**Affiliations:** Department of Gastric and Colorectal Surgery, General Surgery Center, The First Hospital of Jilin University, Changchun, China

**Keywords:** inflammatory bowel disease, causal relationship, Mendelian randomization, Crohn’s disease, ulcerative colitis

In the published article, there were some errors. Because of our carelessness and inattention when we first undertook manuscript writing, we made these errors.

A correction has been made to **Abstract**, *Results.* This sentence previously stated:

“Subsequent meta-integration of the two datasets provided evidence of solid causal associations between 20 immune phenotypes and IBD and its subtypes. Nominal causal associations were also identified in the remaining six immune phenotypes and IBD and its subtypes.”

The corrected sentence appears below:

“Subsequent meta-integration of the two datasets provided evidence of solid causal associations between 18 immune phenotypes and IBD and its subtypes. Nominal causal associations were also identified in the remaining eight immune phenotypes and IBD and its subtypes.”

A correction has also been made to **Abstract**, *Conclusion*. This sentence previously stated:

“Our study confirms causal solid associations between 20 immune phenotypes and IBD, thus guiding future clinical studies.”

The corrected sentence appears below:

“Our study confirms causal solid associations between 18 immune phenotypes and IBD, thus guiding future clinical studies.”

Finally, a correction has also been made to **Discussion**, paragraph 1. This sentence previously stated:

“The results of the study indicate that 24 immune phenotypes may be causally linked to IBD and its different subtypes. Combining the results from two datasets, we discovered that 20 immune phenotypes remain strongly causally linked to IBD and its subtypes.”

The corrected sentence appears below:

“The results of the study indicate that 26 immune phenotypes may be causally linked to IBD and its different subtypes. Combining the results from two datasets, we discovered that 18 immune phenotypes remain strongly causally linked to IBD and its subtypes.”

The authors apologize for these errors and state that this does not change the scientific conclusions of the article in any way. The original article has been updated.

