# Supplementary material for: The immune factors have complex causal regulation effects on inflammatory bowel disease
Source: Front Immunol. 2024 Jan 9;14:1322673. doi: 10.3389/fimmu.2023.1322673 (PMC10803565; doi:10.3389/fimmu.2023.1322673)
Supplement: Supplementary file 9 [file DataSheet_3.docx]

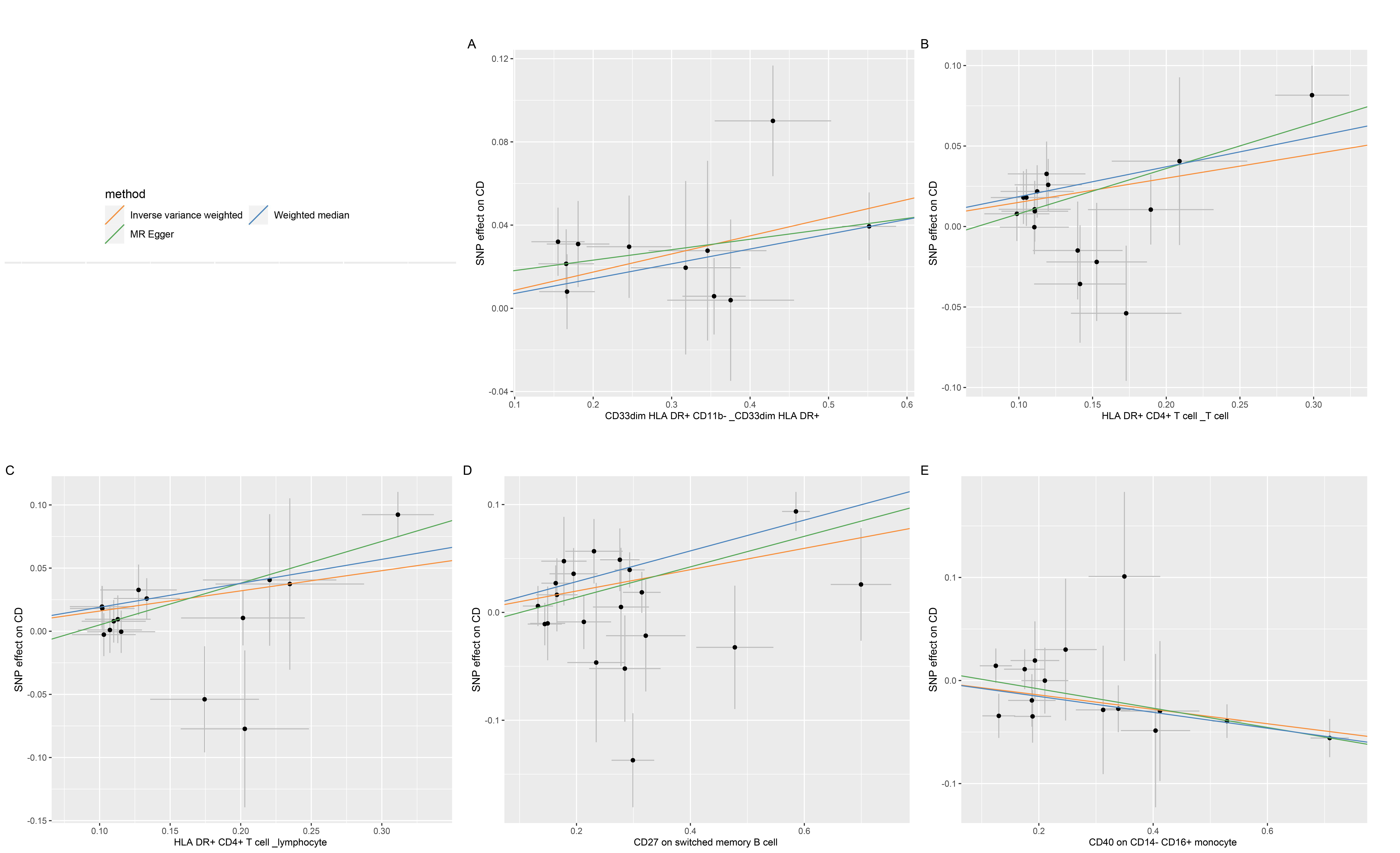


**Figure 1:** Scatter plots for the causal association between immune phenotypes and CD in the IEU dataset. IEU, MRC Integrative Epidemiology Unit; HLA, human leukocyte antigen; IBD, Inflammatory Bowel Disease.


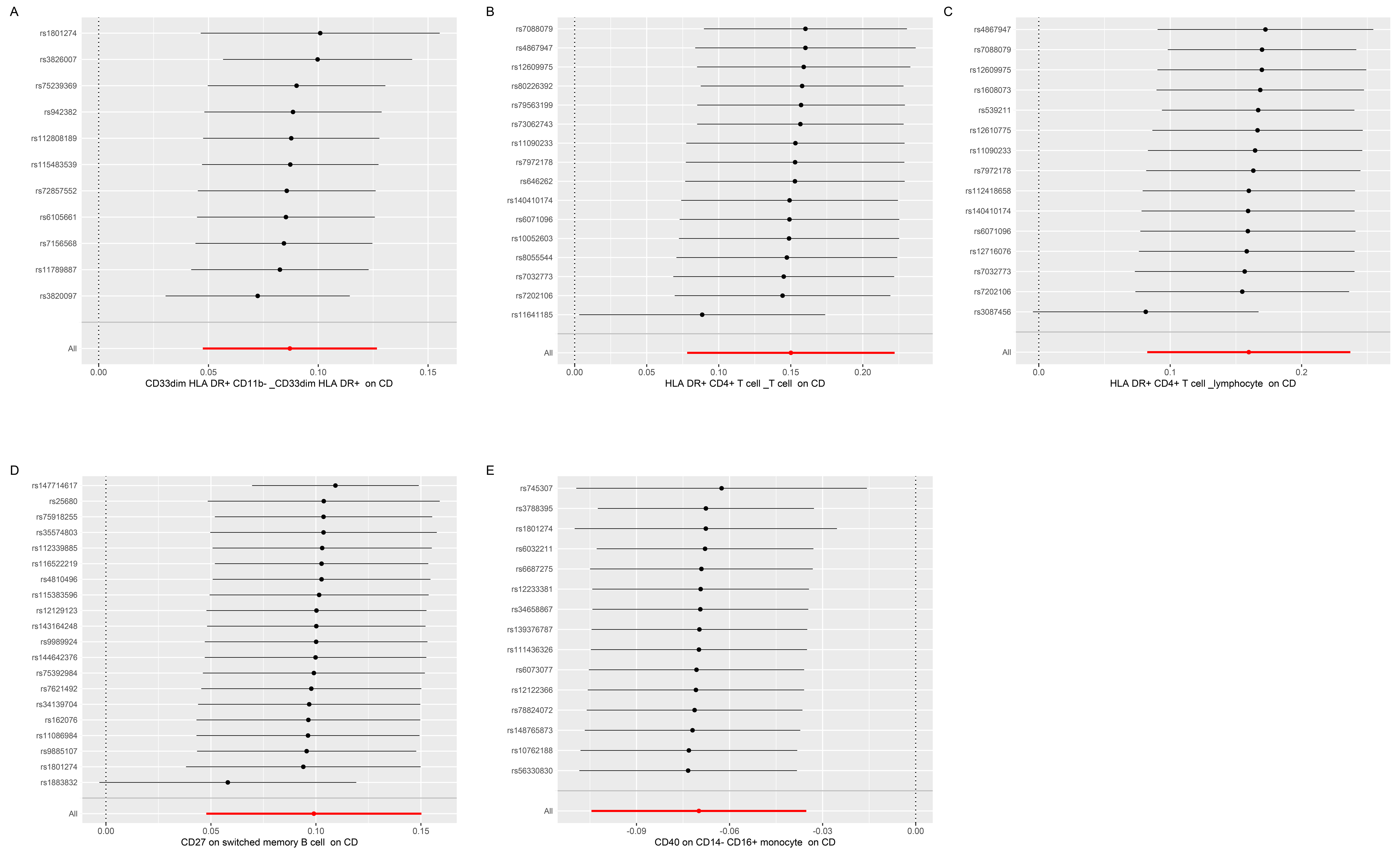


**Figure 2:** Leave-one-out plots for the causal association between immune phenotypes and CD in the IEU dataset. IEU, MRC Integrative Epidemiology Unit; HLA, human leukocyte antigen; IBD, Inflammatory Bowel Disease.


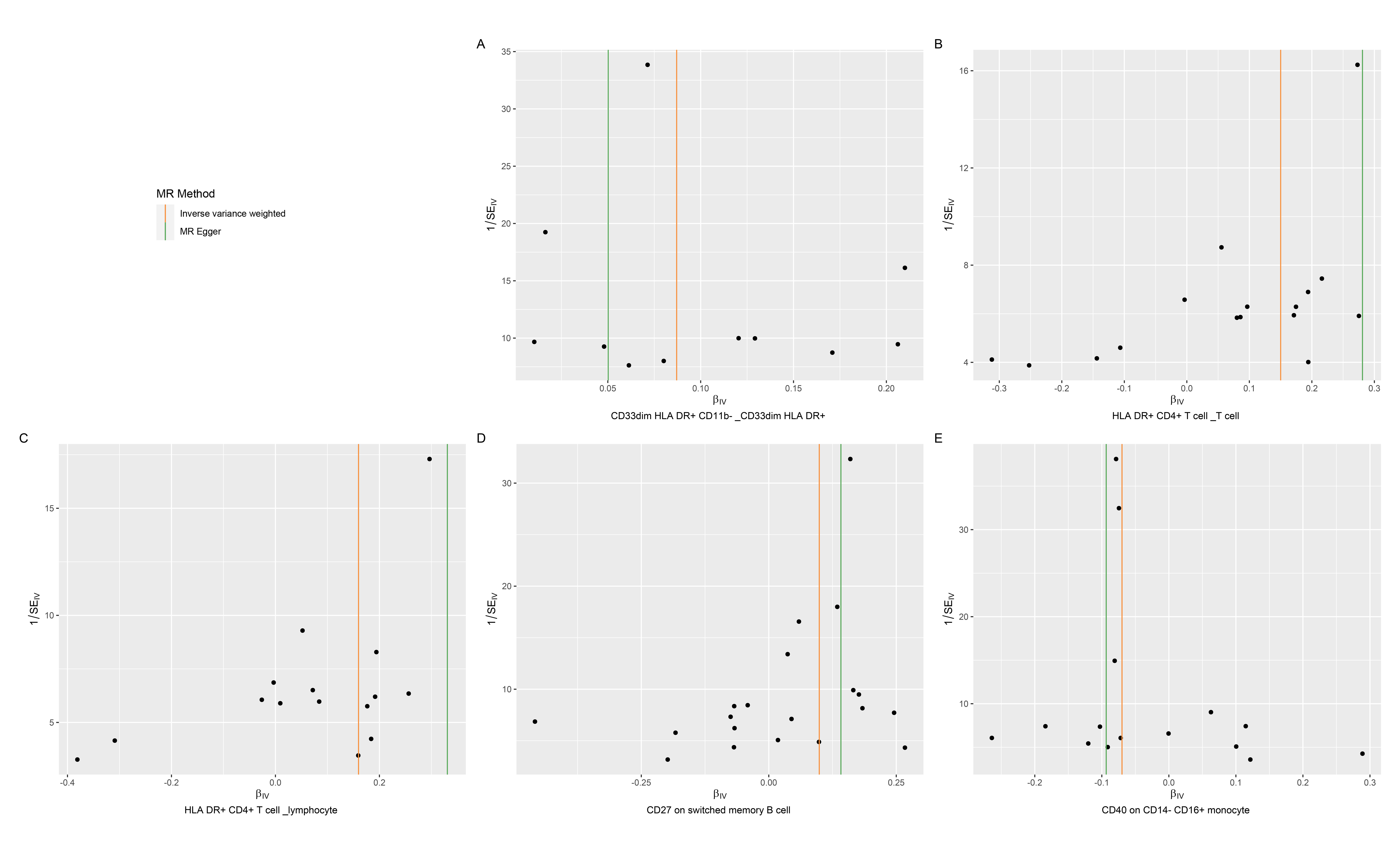


**Figure 3:** Funnel plots for the causal association between immune phenotypes and CD in the IEU dataset. IEU, MRC Integrative Epidemiology Unit; HLA, human leukocyte antigen; IBD, Inflammatory Bowel Disease.
