# Supplementary material for: The immune factors have complex causal regulation effects on inflammatory bowel disease
Source: Front Immunol. 2024 Jan 9;14:1322673. doi: 10.3389/fimmu.2023.1322673 (PMC10803565; doi:10.3389/fimmu.2023.1322673)
Supplement: Supplementary file 11 [file DataSheet_5.docx]

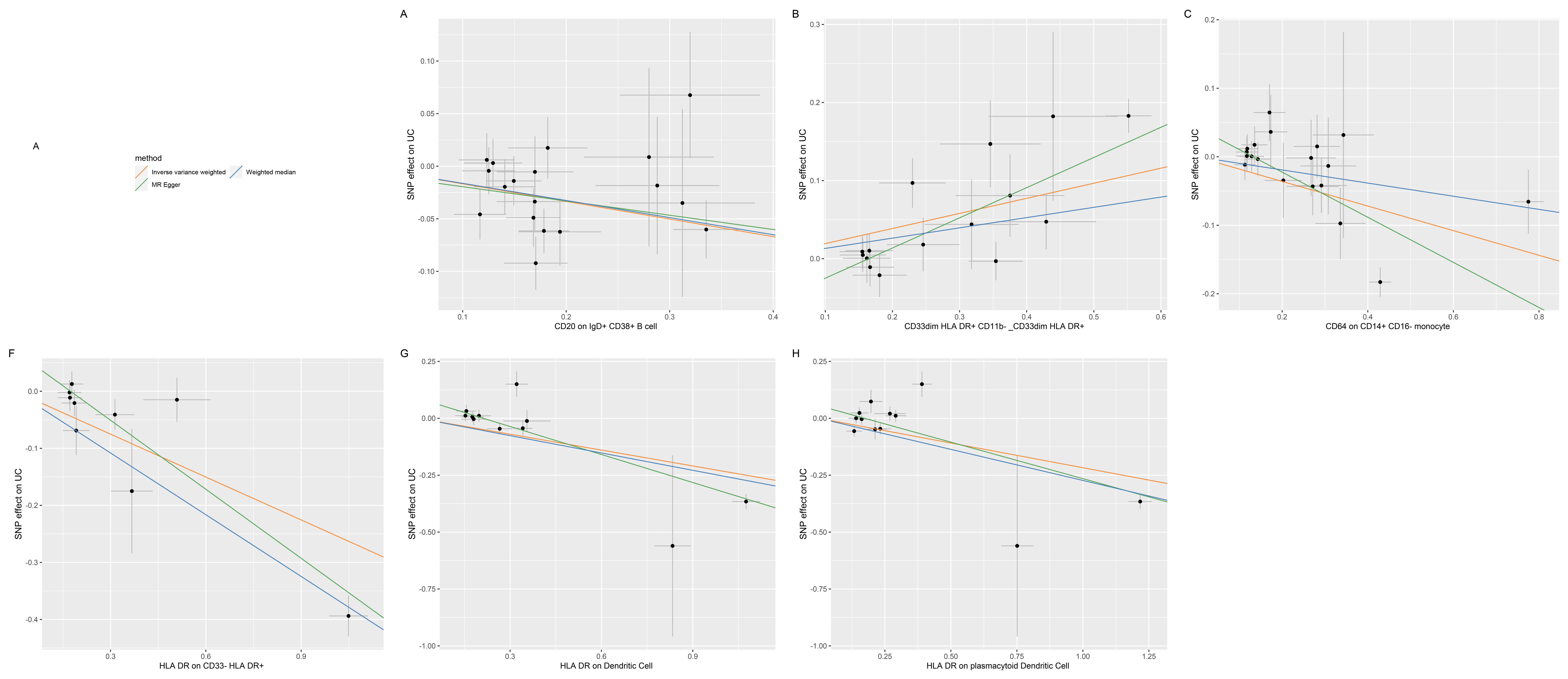


**Figure 1:** Scatter plots for the causal association between immune phenotypes and UC in the IBDGC dataset. IBDGC, The International Inflammatory Bowel Disease Genetics Consortium; IBD, Inflammatory Bowel Disease; HLA, human leukocyte antigen.


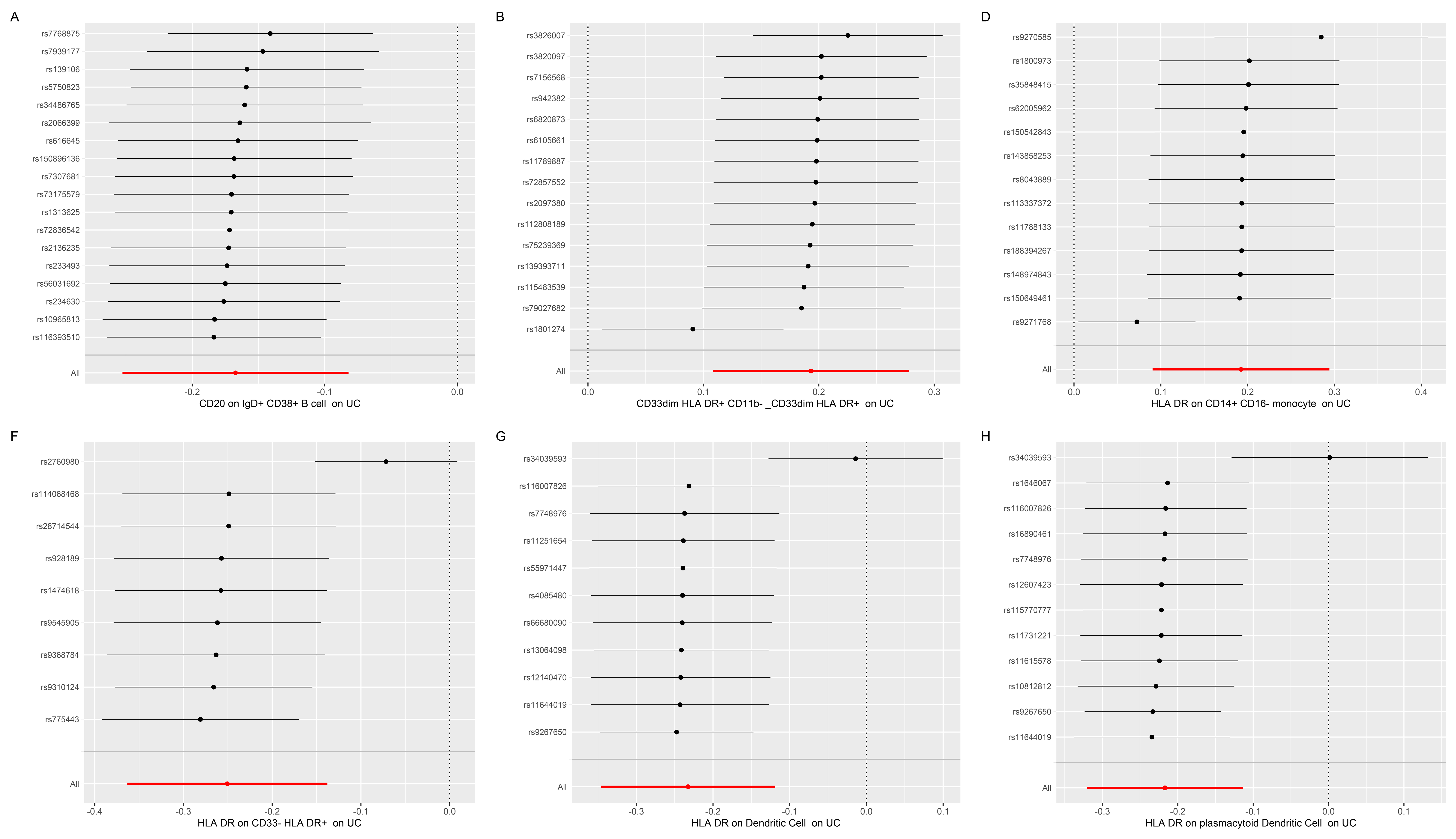


**Figure 2:** Leave-one-out plots for the causal association between immune phenotypes and UC in the IBDGC dataset. IBDGC, The International Inflammatory Bowel Disease Genetics Consortium; IBD, Inflammatory Bowel Disease; HLA, human leukocyte antigen.


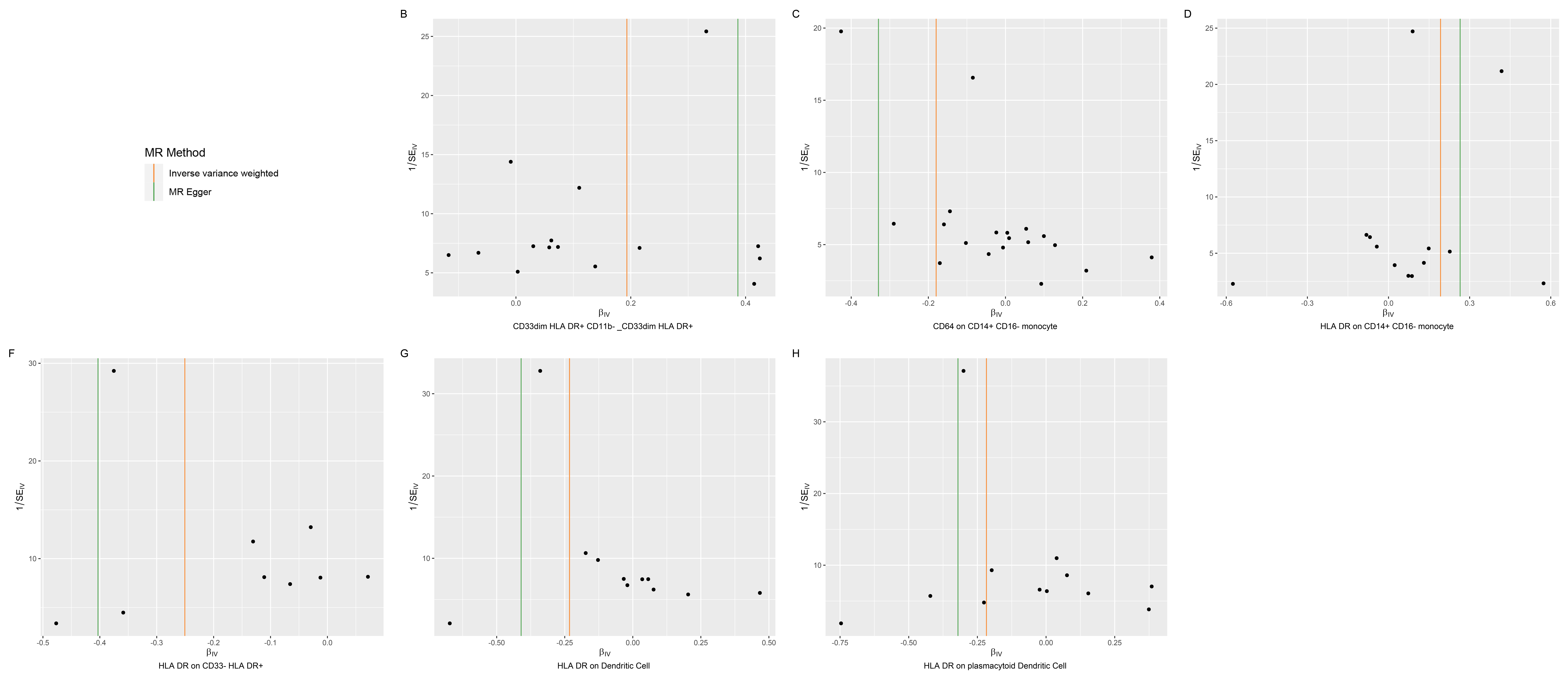


**Figure 3:** Funnel plots for the causal association between immune phenotypes and UC in the IBDGC dataset. IBDGC, The International Inflammatory Bowel Disease Genetics Consortium; IBD, Inflammatory Bowel Disease; HLA, human leukocyte antigen.
